# Effect of Chemical Refining on the Reduction of β-Carboline Content in Sesame Seed Oil

**DOI:** 10.3390/molecules28114503

**Published:** 2023-06-01

**Authors:** Lili Shi, Ziyu Cui, Wei Liu

**Affiliations:** 1 College of Food Science and Technology, Henan University of Technology, Lianhua Street 100, Zhengzhou 450001, China; shililislla@163.com (L.S.); cuiziyu155@163.com (Z.C.); 2 National Engineering Laboratory for Wheat & Corn Further Processing, Henan University of Technology, Lianhua Street 100, Zhengzhou 450001, China

**Keywords:** adsorbent, β-carbolines, harman, norharman, sesame seed oil

## Abstract

β-carbolines (harman and norharman) are potentially mutagenic and have been reported in some vegetable oils. Sesame seed oil is obtained from roasted sesame seeds. During sesame oil processing, roasting is the key procedure to aroma enhancement, in which β-carbolines are produced. Pressed sesame seed oils cover most market share, while leaching solvents are used to extract oils from the pressed sesame cake to improve the utilization of the raw materials. β-carbolines are nonpolar heterocyclic aromatic amines with good solubility in leaching solvents (*n*-hexane); therefore, the β-carbolines in sesame cake migrated to the leaching sesame seed oil. The refining procedures are indispensable for leaching sesame seed oil, in which some small molecules can be reduced. Thus, the critical aim is to evaluate the changes in β-carboline content during the refining of leaching sesame seed oil and the key process steps for the removal of β-carbolines. In this work, the levels of β-carbolines (harman and norharman) in sesame seed oil during chemical refining processes (degumming, deacidification, bleaching and deodorization) have been determined using solid phase extraction and high performance liquid chromatography-mass spectrometry (LC-MS). The results indicated that in the entire refining process, the levels of total β-carbolines greatly decreased, and the adsorption decolorization was the most effective process in reducing β-carbolines, which might be related to the adsorbent used in the decolorization process. In addition, the effects of adsorbent type, adsorbent dosage and blended adsorbent on β-carbolines in sesame seed oil during the decolorization process were investigated. It was concluded that oil refining can not only improve the quality of sesame seed oil, but also reduce most of the harmful β-carbolines.

## 1. Introduction

Heterocyclic aromatic amines (HAAs) are a class of polycyclic aromatic compound containing N-heterocycles, which are produced by the reaction of free amino acids, sarcosine, creatinine and sugars under high temperature. HAAs have carcinogenic and mutagenic activities [1]. So far, more than 30 HAAs have been identified in various foods [2]. β-carbolines, mainly including harman (1-methyl-9H-pyrido[3,4-b]indole) and norharman (9H-pyrido[3,4-b]indole), are very easy to generate in thermal food processing, such as baking and roasting. These two substances are not mutagenic by themselves, but when coexisting with aniline, o-toluidine or other HAAs, they will cause strong carcinogenicity [3]. Animal studies have also shown that harman and norharman affected the physiological behavior of animals, mainly because they bind to certain sites in the liver and brain of mice [4,5]. In addition, harman and norharman can poison some nerves and inhibit some key enzymes.

Over the past few decades, β-carbolines (harman and norharman) have been detected in many processed foods, including thermally processed meats (e.g., pork), coffee products and tobacco smoke [6,7,8]. At present, more attention has been paid to HAAs in foods of animal origin, but relatively less to HAAs in foods of plant origin. Wojtowicz et al. revealed that the β-carboline contents in chicory coffee increased rapidly (up to 25-fold) after roasting at 160 °C [9]. Theoretically, plant-protein-based foods can also produce HAAs after roasting at high temperatures. Coffee beans contain only about 13% protein, but high-temperature roasting can produce HAAs. Because of the roasting process of oil seeds (e.g., sesame seed, peanut), some vegetable oils (e.g., sesame seed oil) may be considered as the main contributor to β-carboline compounds. There are limited reports on the levels of β-carbolines (harman and norharman) in edible oils, especially in sesame seed oil and other flavored vegetable oils. Recently, Chang et al. showed that the levels of harman and norharman in sesame seed oils were 168 μg/kg and 162 μg/kg, respectively, while HAAs were not detected in other vegetable oils (e.g., linseed oil, soybean oil) [10]. In addition, Zhang et al. reported that the levels of harman and norharman in sesame seed oils were 453 μg/kg and 276 μg/kg, respectively, which were much higher than those in sunflower oil, canola oil and peanut oil [11]. Our group has analyzed commercial sesame seed oils (pressed fragrant sesame oils, ground fragrant sesame oils, cold-pressed sesame oils) produced by different processes and found that most of sesame oil samples contained both harman and norharman [12].

**Figure 1:**
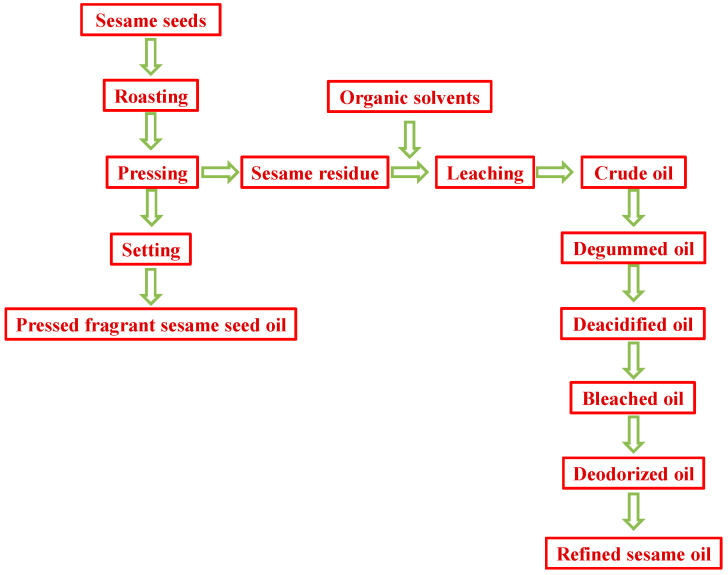
The processing procedure of sesame seed oils.

Sesame seed oil (sesamum indicum) is a traditional edible oil in China and other Asian regions. There are four kinds of sesame seed oils in China, including pressed fragrant sesame seed oil, ground fragrant sesame seed oil, cold-pressed sesame seed oil and refined sesame seed oil. Pressed fragrant sesame oil is generally produced using mechanical pressing after high-temperature roasting. The production of pressed fragrant sesame seed oil leads to a large amount of sesame meal, which can be used to produce refined sesame seed oil though solvent extraction and the subsequent refining process (Figure 1). Whereas β-carbolines (harman and norharman) are non-polar HAAs with good solubility in leaching solvents (e.g., hexane), the β-carbolines in sesame meal migrate into the leached sesame oil. It has been shown that chemical refining can reduce most of the minor components of vegetable oils (e.g., soybean oil) such as free fatty acids, vitamin E, phytosterols and other small molecule compounds [13,14,15]. Therefore, it is meaningful to investigate the level of HAAs (β-carbolines) in the refining process of sesame seed oil.

Oil refining usually consists of degumming, deacidification, decolorization and deodorization steps, of which decolorization is one of the important processes in edible oil refining. Some studies have reported that solid adsorbents (e.g., clay and activated carbon) used in the decolorization process can bind to small molecules (e.g., pigments) in oils via physical adsorption [16,17]. Recently, it has been shown that decolorization is a key process to reduce some harmful compounds (e.g., BaP) [18,19,20]. Thus, the effect of sorbents on the level of HAAs (β-carbolines) in the oil-refining process can also provide a possible method for the removal of HAAs. In this work, the level of β-carbolines (harman and norharman) in crude sesame oil and oils during refining processes (degumming, deacidification, bleaching and deodorization) has been studied and the effect of the chemical refining process on the level of harman and norharman in sesame seed oils has been discussed. Importantly, the effect of adsorbent type, adsorbent dosage and composite adsorbent on the removal of β-carbolines from sesame seed oil during the decolorization process was investigated as well. This work will provide new insight into the removal of HAAs (β-carbolines) from edible oils.

## 2. Results and Discussion

### 2.1. Effects of Sesame Seed Oil Refining Process on the Content of β-Carboline Compounds

**Figure 2:**
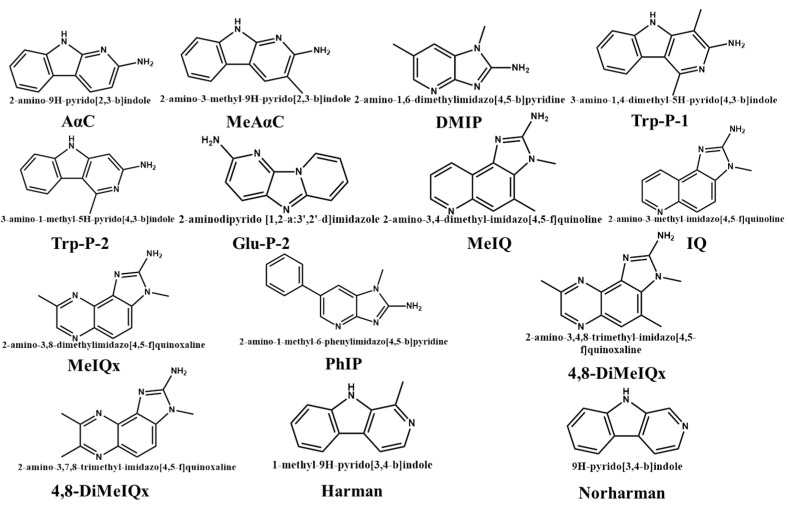
The chemical structure of 14 heterocyclic aromatic amines.

To explore the changes in HAA content during the refining process of sesame seed oil, the level of HAAs in sesame crude oil and oils during refining processes (degumming, deacidification, bleaching and deodorization) have been detected using LC-MS. A flow chart of the refining process of sesame seed oil is shown in Figure 1. The chemical structures of the 14 heterocyclic aromatic amines are shown in Figure 2.

**Figure 3:**
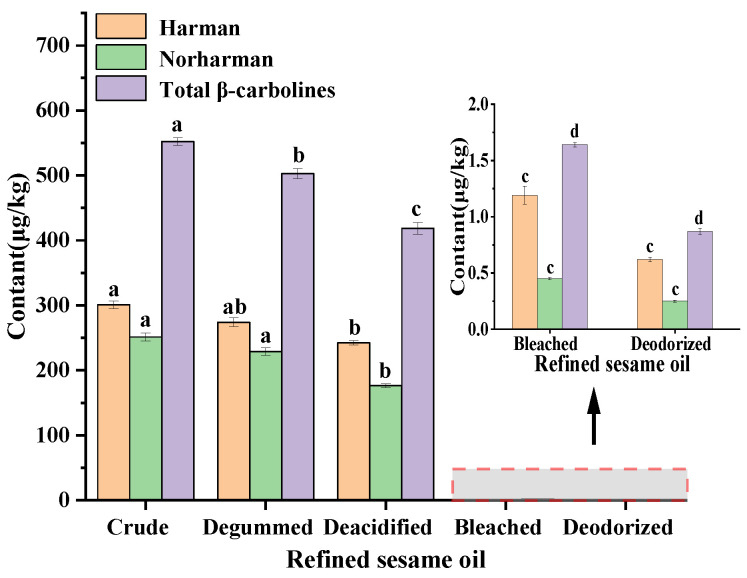
Changes in β-carboline content during the refining of sesame seed oil. Note: The decolorizer used in the decolorization process was clay (3% of oil weight). Means (n = 3) across all samples without a common letter differ significantly (*p* < 0.05).

The levels of HAAs in sesame crude oil, degumming oil, deacidification oil, bleached oil and deodorized oil were determined, respectively. The results showed that the HAAs in crude sesame seed oil were mainly β-carbolines (harman and norharman) [10,12] (Table 1). The levels of total β-carbolines (harman and norharman) decreased from 552.34 μg/kg to 502.69 μg/kg and 418.50 μg/kg during degumming and deacidification of sesame seed oil, respectively (Figure 3). This may be due to the formation of colloids in the hydration degumming process. The soap formed in the deacidification process adsorbed some small molecule compounds (e.g., harman and norharman) in sesame seed oil, but the ability to remove β-carbolines was limited [21,22]. Notably, the bleaching step could significantly reduce β-carbolines (harman and norharman) in sesame seed oil from 418.50 μg/kg to 1.64 μg/kg, which might be attributed to the absorbents (e.g., activated clay and activated carbon) used in this step [23]. Some studies reported that solid adsorbents (e.g., activated clay and activated carbon) could bind to small molecules (e.g., pigmented substances) in oils via physical adsorption [24], which also contributed to the removal of β-carboline compounds. Decolorization is one of the important processes in the refining of edible oils. In addition to removing pigments and improving the appearance of the oil, it can also decrease the content of free fatty acids, phospholipids, peroxides, carbonyl compounds and other small molecular compounds (e.g., polycyclic aromatic hydrocarbons) in oils [23]. Thus, the decolorization process is a key step for the effective reduction of harman and norharman content in sesame seed oil.

### 2.2. Effect of Different Types of Adsorbents on the Removal of β-Carbolines from Sesame Seed Oil

**Figure 4:**
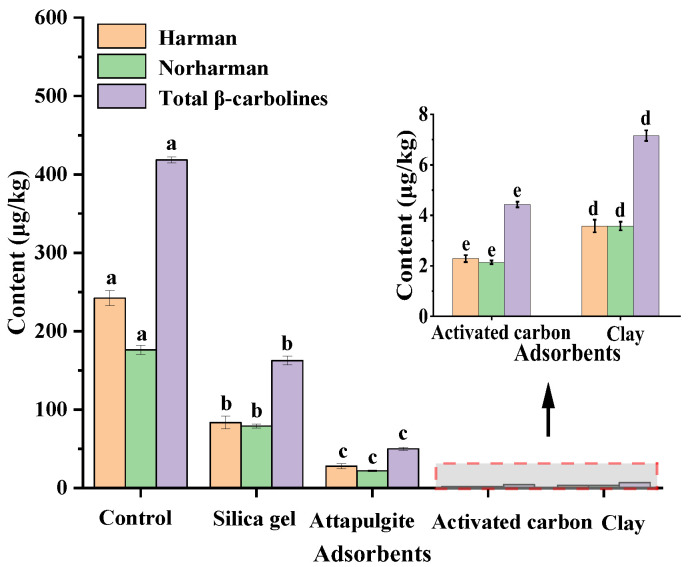
Effects of different types of decolorizing agents on the removal of β-carbolines in sesame seed oil. Note: The control group was deacidified oil, and the dosage of decolorizers was 1% of the oil weight. Means (n = 3) across all samples without a common letter differ significantly (*p* < 0.05).

To investigate the effect of adsorbent type on the removal of the β-carbolines (harman and norharman) in sesame seed oil, different kinds of decolorizers, including silica gel, attapulgite, activated carbon and clay, were used in adsorption decolorization experiments. The results showed that the four adsorbents were all effective in the removal of β-carbolines (harman and norharman) from sesame seed oil (Figure 4). When the adsorbents silica gel, attapulgite, activated carbon and clay were added to the sesame deacidification oil for decolorization, respectively, the content of β-carbolines decreased from 418.50 μg/kg to 162.64 μg/kg, 50.04 μg/kg, 4.43 μg/kg and 7.16 μg/kg, respectively.

In general, activated carbon and clay were more effective for the removal of β-carbolines (harman and norharman). The reason may be that activated carbons have hydrophobic graphene layers, which can interact readily with non-polar compounds (e.g., harman and norharman) [25]. Clay had a large specific surface area, high activity and strong adsorption capacity for minor compounds (e.g., harman and norharman) in sesame seed oil [26]. However, the adsorption capacity of attapulgite and silica gel for harman and norharman was relatively inferior. The possible reason was that many adsorption active points on the pore surface of attapulgite preferentially adsorbed polar substances and adsorbed non-polar compounds less (e.g., harman and norharman) [27]. Silica gel was a strong polar adsorbent, which was favorable for adsorbing high polar compounds [28].

### 2.3. Effect of Adsorbent Dosage on the Removal of β-Carbolines from Sesame Seed Oil

**Figure 5:**
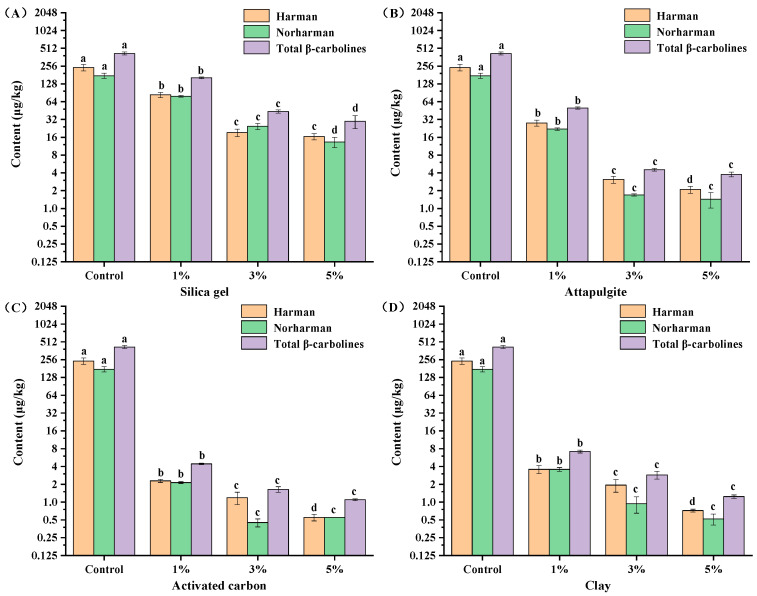
Effect of adsorbent dosage on the removal of β-carbolines from sesame seed oil. Note: (**A**–**D**) represent the adsorbents used were silica gel, attapulgite, activated carbon and clay respectively. The control group was deacidified oil, and the dosage of decolorizers was the mass fraction of the oil weight. Means (n = 3) across all samples without a common letter differ significantly (*p* < 0.05).

The removal effect of β-carbolines (harman and norharman) in the decolorization process of sesame seed oil was also related to the amount of adsorbents. To investigate the effect of adsorbent dosage on β-carbolines in sesame seed oil, the decolorization of sesame seed oil was carried out at different adsorbent dosages (1%, 3%, 5%). The results showed that the total β-carbolines (harman and norharman) in sesame seed oil gradually decreased as the amount of adsorbents (silica gel, attapulgite, activated carbon and clay) increased from 1% to 5% (Figure 5). When the bleaching agents were silica gel, attapulgite, activated carbon and clay, with the dosage increasing from 1% to 5%, the total β-carbolines (harman and norharman) in bleached oil decreased from 162.64 μg/kg to 29.76 μg/kg, 50.04 μg/kg to 3.78 μg/kg, 4.43 μg/kg to 1.10 μg/kg and 7.16 μg/kg to 1.24 μg/kg, respectively (Figure 5). The above results suggested that 5% clay and 5% activated carbon were more effective in decreasing the content of harman and norharman in sesame seed oil. Among them, when activated carbon and clay were added at 5% of oil weight, the contents of harman in decolorized oil were 0.55 μg/kg and 0.72 μg/kg, respectively, and the contents of norharman were 0.55 μg/kg and 0.52 μg/kg, respectively (Figure 5C,D).

### 2.4. Effects of Different Types of Activated Carbons on the Removal of β-Carbolines from Sesame Seed Oil

**Figure 6:**
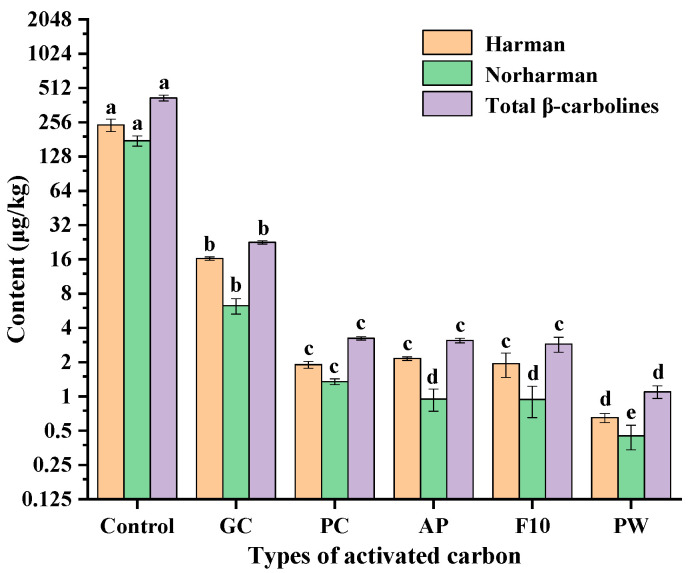
Effects of different types of activated carbons for the removal of β-carbolines from sesame seed oil. Note: The control group was deacidified oil, and amount of activated carbons was 1% of the oil weight. The symbols for each activated carbon are as follows: GC, granular type activated carbon made from coconut shell; PC, powdered type activated carbon made from coconut shell; AP, F10, powdered type activated carbon made from peat; PW, powdered type activated carbon made from wood. Means (n = 3) across all samples without a common letter differ significantly (*p* < 0.05).

Activated carbon is made from a wide range of raw materials, generally from coal, peat, wood powder, coke from coconut shells and other raw materials through carbonization and activation processing [26]. Thus, different types of activated carbons (wood powder, coconut shell powder, coconut shell particles, coal, etc.) were carried out for the adsorption and decolorization process of sesame oil. Since the capacity of activated carbon is affected by its specific surface area, pore structure and surface functional groups, etc. [29], different types of activated carbons had different adsorption capacities for β-carbolines (harman and norharman) in sesame seed oil. Figure 6 shows the contents of β-carbolines in sesame seed oil after decolorization by different types of activated carbons (GC, PC, AP, F10 and PW). PW significantly decreased the harman and norharman levels in sesame seed oil compared with those of the control (418.50 μg/kg) to 1.10 μg/kg, followed by F10 (2.88 μg/kg), AP (3.10 μg/kg), PC (3.24 μg/kg) and GC (22.52 μg/kg). Among the activated carbons, GC was less effective in the removal of β-carbolines from sesame seed oil, probably because it is a granular activated carbon and had relatively less contact area with sesame seed oil during decolorization. The surface properties of activated carbon such as porosity and specific surface area differ based on the raw materials used [30]. Lee et al. demonstrated that powdered activated carbon made from wood had a larger specific surface area than powdered activated carbon made from other raw materials [31]. Thus, the powdered activated carbon PW was more effective in reducing the β-carboline level of the sesame seed oil compared with activated carbon F10, AP and PC.

### 2.5. Removal of β-Carbolines from Sesame Seed Oil Using Blended Decolorizers

**Figure 7:**
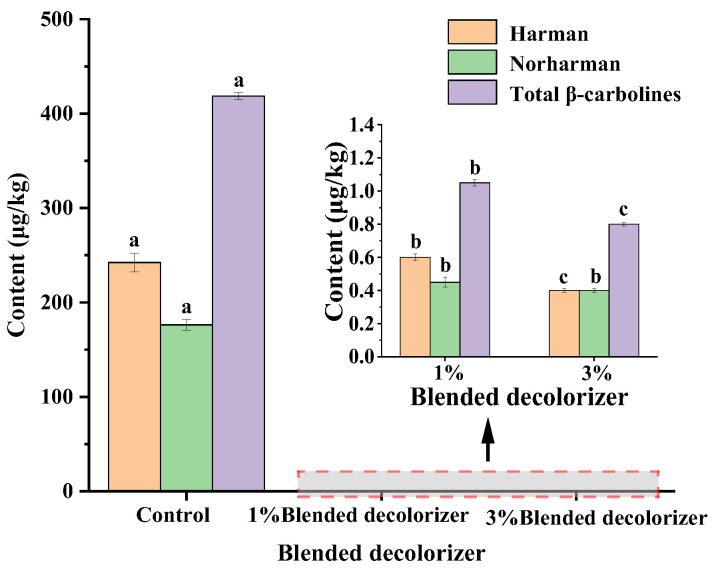
Effects of blended decolorizer on the removal of β-carbolines from sesame seed oil. Note: The control group was deacidified oil, and the dosage of blended decolorizer was the mass fraction of the oil weight. The blended decolorizer was a 1:1 mass ratio of clay and activated carbon (PW). Means (n = 3) across all samples without a common letter differ significantly (*p* < 0.05).

In general, in the process of oil decolorization, too much clay results in the acid value rising and an earthy smell, while too much activated carbon causes large oil loss. In the present study, activated carbon compounded with a clay mixture was usually used for decolorization in the grease decolorization process [19,32]. It not only improved the decolorization capacity and reduced the risky substances (e.g., harman and norharman), but also decreased the adsorbent dosage and loss of oil. Therefore, the adsorption decolorization experiments were carried out by using a blended decolorizer of white clay and activated carbon (PW) with a mass ratio of 1:1, and the amount of decolorizer added was 1% and 3% of the oil weight, respectively. The results showed that when adding 1% and 3% blended decolorizer for the decolorization process, respectively, the contents of β-carboline compounds decreased from 418.50 μg/kg to 1.05 μg/kg and 0.80 μg/kg, respectively (Figure 7). The blended decolorizer was more capable of removing heterocyclic aromatic amines (HAAs) than a single type of adsorbent. Meanwhile, the introduction of activated carbon (PW) could improve the decolorization performance of clay for edible oils and also effectively reduce the content of risk substances (e.g., harman and norharman). A possible reason was that clay preferentially removed impurities (e.g., pigments) from sesame seed oil, preserving the adsorption capacity for the removal of β-carbolines by activated carbon (PW). Thus, a blended decolorizer consisting of activated carbon and clay could effectively remove HAAs from sesame seed oil while meeting the process requirements for the decolorization of edible oils.

### 2.6. Changes in Basic Physicochemical Properties of Sesame Seed Oil Refining Process

To evaluate the changes in physicochemical indexes during the refining process of sesame seed oil, the acid value (AV), peroxide value (POV) and color of sesame seed oil were determined (Table 2 and Table 3).Therein, the AV and POV are important indicators of basic edible oil quality, which are closely related to oil stability. The AV and POV of the leached sesame seed oil (crude oil) were 1.72 mg/g and 0.037 mmol/kg, respectively. The AV of sesame seed oil decreased continuously in the process of chemical refining, probably due to the adsorption of small amounts of free fatty acids by the oil soap horns during the deacidification process. The AV and POV in the final refined sesame seed oil were 0.28 mg/g and 0.003 mmol/kg, respectively, which reached the quality index of first-grade refined sesame seed oil. The deodorization process removes most of the small molecules and unstable compounds (e.g., peroxide compounds). Therefore, except for improving the quality of sesame seed oil, refining could reduce most of the harmful substances as well.

**Figure 8:**
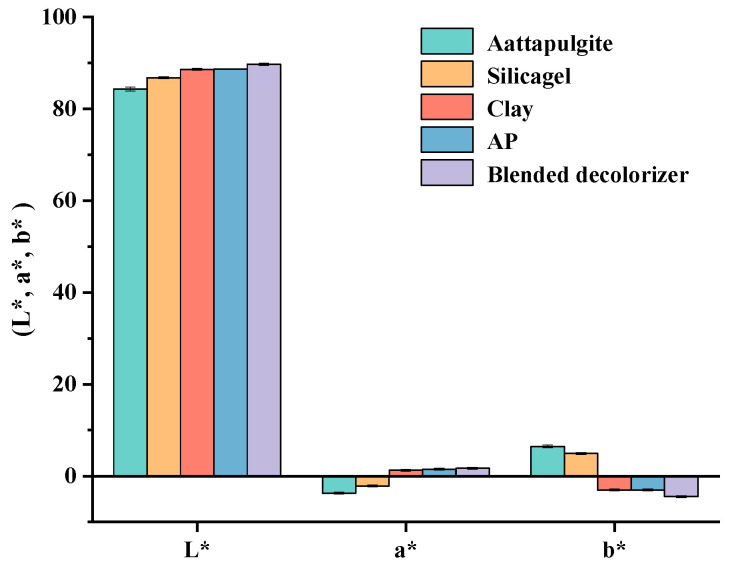
The color of sesame seed oil after decolorization with different decolorizers. Note: The dosage of decolorizers was 3% of the oil weight. PW, powdered type activated carbon made from peat. The blended decolorizer was a 1:1 mass ratio of clay and activated carbon (PW).

Changes to the color of sesame seed oil during the refining process were investigated. L*, a* and b* denote the chromaticity value of the object color, where any color has a unique coordinate value for the color space coordinates. L* denotes brightness (black and white), a* denotes green to red and b* denotes blue to yellow. Crude sesame oil exhibited lower L* and a* values and higher b* values (Table 3, Figure 8). This meant that crude sesame oil was darker, greener and more yellow than the refined sesame seed oil. The color formation in sesame seed oil was probably due to nonenzymatic browning (Maillard reaction), which occurred during the roasting of sesame seeds [33]. Some studies have demonstrated that roasting increased the color of dark and yellow units as well as caused the production of green pigments, especially chlorophyll [34,35,36]. In contrast, pigments could be removed during the degumming, deacidification and decolorization processes, so that the color of sesame seed oil was clear and transparent with less green and yellow during refining (L* and a* values gradually increase, while b* values gradually decrease). Notably, compared with other decolorizers, the color of sesame seed oil after decolorization with 3% blended decolorizer was clear and transparent with less yellow (L*: 89.58; a*: 1.72; b*: −4.47). This means that the removal of pigments from sesame seed oil is related to the adsorbent type and dosage.

## 3. Materials and Methods

### 3.1. Materials

White sesame seed was purchased from a local supermarket. The five adsorbents of activated carbons used in the present study were as follows: powdered type activated carbon made from peat (AP, F10), powdered type activated carbon made from wood (PW), powdered type activated carbon made from coconut shell (PC) and granular type activated carbon made from coconut shell (GC). F10, PW, PC and GC were purchased from Xin Sen Carbon Co., Ltd. (Nanping, China). AP was obtained from Aladdin Biochemical Technology Co., Ltd. (Shanghai, China). Acetonitrile (HPLC grade) was purchased from Thermo Fisher Scientific (Shanghai, China). Ammonium hydroxide and hydrochloric acid (HPLC grade) were obtained from Kemiou Chemical Reagent Co., Ltd. (Tianjin, China). Methyl alcohol, acetic acid and *n*-hexane were of HPLC grade, and other chemicals were of analytical reagent grade. Oasis MCX solid-phase extraction cartridge (150 mg, 6 mL) was purchased from Waters (Milford, CT, USA). Additionally, the water used was Wahaha purified water purchased from a local supermarket. The standards AαC, MeAαC, DMIP, Trp-P-1, Trp-P-2, Glu-P-2, MeIQ, MeIQx, IQ, PhIP, 4,8-DiMeIQx, 7,8-DiMeIQx were purchased from Toronto Research Chemicals (Toronto, ON, Canada). Harman, Norharman and 4,7,8-TriMeIQx were purchased from Alta scientific (Tianjin, China).

### 3.2. Methods

#### 3.2.1. Roasting Treatment and Extraction of Oil from Sesame Seeds

The sesame seeds were roasted using an oven at 220 °C for 20–30 min. The roasted sesame seeds were cooled naturally at room temperature and then fed into an oil press to obtain sesame seed oil and sesame cake. The sesame cake was extracted with *n*-hexane three times. The obtained mixture (oil–hexane) was processed under reduced pressure to remove the solvent (*n*-hexane), and the leached sesame seed oil for the following experiments was afforded.

#### 3.2.2. Hydration Degumming

The above-prepared oil sample was stirred in a water bath at 60 °C for 30–40 min. The distilled water was heated to the oil temperature and added to the above oil sample. The water should be sprayed evenly, and the added water was 2~3 times the phosphorus content. At the end of hydration, a reduction in stirring speed was beneficial for flocculation separation. After hydration, the degummed oil was obtained via centrifugation.

#### 3.2.3. Alkali Refining Deacidification

The specified concentration of NaOH was added to the obtained oil sample to neutralize the free fatty acids and additional NaOH was infused to ensure the complete formation of soaps. This process was initially carried out at 30 °C with stirring. Once the NaOH was added, the temperature was immediately raised to the specified temperature, maintaining the temperature for the required time. Then, the soaps were separated via centrifugation at 5000 r/min. To remove the remnants of the soap and reagents dissolved in the treated oil, the oil obtained in the previous stages was washed with 90 °C pure water three times successively. Finally, the deacidified oil was obtained by heating under reduced pressure to remove the washing water.

#### 3.2.4. Adsorption Decolorization

The removal of HAAs using the oil bleaching procedure was conducted using different adsorbents. The different amounts of adsorbent (1%, 3%, 5%) adopted were added to the prepared oil sample, and the mixture was kept at 80 °C for 30 min by stirring in a vacuum atmosphere. Then, the adsorbent was removed under filtration and the bleached oil was obtained.

#### 3.2.5. Distillation Deodorization

The deodorization was a type of distillation process to remove volatile compounds and heat-instable pigments. Deodorization parameters were set as follows: 240 °C of deodorization temperature, 80 Pa of deodorization pressure and 60 min of deodorization time. Then, the oil samples was cooled down to room temperature and the deodorized oil was obtained.

#### 3.2.6. Extraction and Purification of HAAs

The extraction of HAAs from oil samples was according to the reference with slight modification [37]. Approximately 2 g of oil samples was placed into a 50 mL centrifuge tube. Then, 10 μL 5 mg/L internal standard working solution (4,7, 8-TriMeIQx) and 10 mL acetonitrile solution containing 1% (volume fraction) acetic acid were added. The mixture was homogenized for 1 min, followed by ultrasound for 10 min and cryogenic centrifugation at −4 °C (10,000 r/min, 10 min). The supernatant was collected into a 50 mL centrifuge tube. The above acetonitrile extraction operation was repeated twice. All extracts were collected together.

The solid phase extraction column Oasis MCX cartridges (150 mg/6 mL) were pre-activated with 10 mL methanol and 10 mL 0.1 mol/L hydrochloric acid/methanol (80:20, *v*/*v*). All extracts were transferred to the MCX column for enrichment and purification. Then, 10 mL water, 10 mL methanol and 10 mL methanol/ammonia/water (25:5:75, *v*/*v*/*v*) were added to the MCX column successively for washing and purification. Finally, 10 mL methanol/ammonia mixed solution (95:5, *v*/*v*) was used for elution and collection. All eluent was collected and dried with nitrogen. A mixture of 10 mL 5% formic acid/acetonitrile (95:5, *v*/*v*) was added to the blow-dried sample and then filtered through a 0.45 μm microporous filter for LC/MS analysis.

#### 3.2.7. LC/MS Analysis of HAAs

The determination of HAAs was performed on an Agilent ZORBAX Eclipse XDB-C18 column (3.5 μm, 150 mm × 2.1 mm) at 35 °C. The gradient elution was carried out with 5% formic acid/5 mM ammonium formate aqueous solution (A) and 5% formic acid/5 mM ammonium formate methanol solution (B) as binary mobile phases at a flow rate of 0.4 mL/min. The gradient elution program was as follows: 0–0.01 min, 5%B; 0.01–1.00 min, 5%B; 1.00–1.10 min, 5–60%B; 1.10–5.00 min, 60–80%B; 5.00–6.00 min, 80–95%B; 6.00–8.00 min, 95%B; 8.00–8.10 min, 95–5%B; 8.10–8.20 min, 5%B; 8.20–10.00 min, adjusting mobile phase balance to initial state. The injection volume was 5 μL. The internal standard method was used to quantify the samples, and the results were analyzed under the standard curve established with 14 HAAs (gradient dilution to 0.10, 0.50, 1.00, 2.50, 5.00, 10.00, 20.00, 25.00 ng/mL).

**Figure 9:**
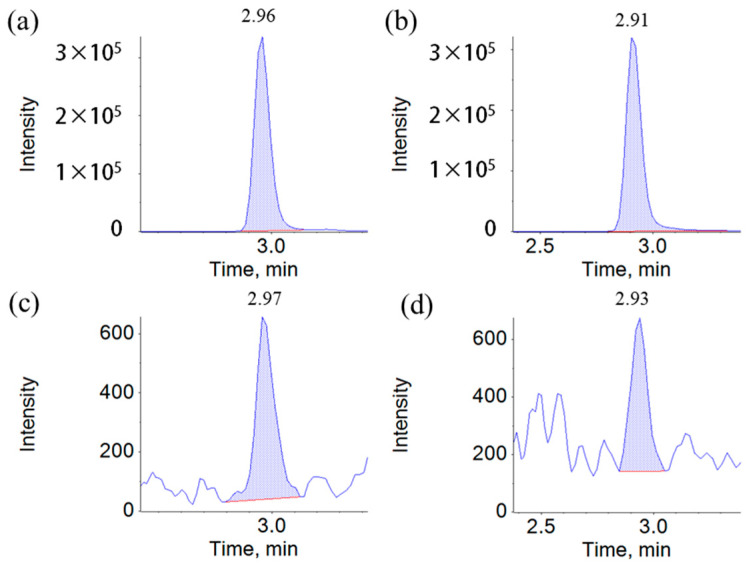
The chromatogram of sample with high content of harman and norharman (**a**,**b**); the chromatogram of sample with low content of harman and norharman (**c**,**d**).

Mass spectrometry was analyzed using positive electrospray ionization (ESI+). The multiple reaction monitoring conditions were automatically optimized. The capillary voltage was 5.5 kV, and the ion source temperature was 550 °C. The qualitative and quantitative characteristic ions and optimized mass spectrometry parameters of 14 heterocyclic aromatic amines (HAAs) and internal standards are shown in Table 4. The chromatograms of harman and norharman in sesame seed oil samples are shown in Figure 9.

#### 3.2.8. Physicochemical Properties of Sesame Seed Oil

The peroxide value (POV) of sesame seed oil was characterized according to GB 5009.229-2016, while the acid value (AV) was analyzed based on the methods described in GB 5009.229-2016. Briefly, for POV, 2.0 g oil sample was titrated with 0.01 mol/L sodium thiosulfate standard solution and POV was expressed as reactive oxygen in mmol/kg of oil. For AV, 2.0 g oil sample was titrated with 0.01 mol/L KOH standard solution and AV was expressed as KOH in mg/g of oil. The sample color was measured using a Chroma Meter CR-400 and the L* value (lightness), a* value (red-green intensity) and b* value (yellow-blue intensity) were measured.

#### 3.2.9. Statistical Analysis

All experiments were carried out in triplicate, and the data were expressed as mean ± standard deviation. Multi Quant software was used for heterocyclic aromatic amines data analysis. Origin Pro software (Origin Lab Co., Northampton, MA, US) was used for charting. The difference between groups was tested using ANOVA and Duncan’s multiple range tests. Means were compared and were considered significant when *p* < 0.05.

## 4. Conclusions

In this paper, the levels of β-carbolines (harman and norharman) in sesame seed oil during the refining process were investigated systematically. The results showed that the levels of the total β-carbolines (harman and norharman) in refined sesame seed oils were much lower than those in pressed fragrant sesame seed oils. Adsorption decolorization was the most effective process to reduce the content of β-carboline compounds, which was related to the adsorbent used in the decolorization process. Three percent blended decolorizer consisting of activated carbon and clay achieved the best results for removing β-carbolines. After refining, the basic physicochemical indexes of sesame seed oil, such as AV, POV and color, reached the quality index of first-grade refined sesame seed oil. It was concluded that oil refining could not only improve the quality of sesame seed oil, but also reduce most of the harmful substances (e.g., β-carbolines). This study will be meaningful to prove that decolorizers are effective to remove small molecule compounds (e.g., nonpolar heterocyclic aromatic amines harman and norharman) in edible oils.

## Figures and Tables

**Table 1:** The contents of 14 HAAs were determined during the refining of sesame seed oil (μg/kg) ^a^.

HAA (μg/kg)	Crude Oil	Degummed	Deacidified	Bleached	Deodorized
AαC	ND ^b^	ND	ND	ND	ND
MeAαC	ND	ND	ND	ND	ND
Trp-P-1	ND	ND	ND	ND	ND
DMIP	ND	ND	ND	ND	ND
Glu-P-2	ND	ND	ND	ND	ND
MeIQ	ND	ND	ND	ND	ND
MeIQx	ND	ND	ND	ND	ND
IQ	ND	ND	ND	ND	ND
PhIP	ND	ND	ND	ND	ND
4,8-DiMeIQx	ND	ND	ND	ND	ND
7,8-DiMeIQx	ND	ND	ND	ND	ND
Harman	301 ± 3	274 ± 2	242 ± 4	1.2 ± 0.3	0.52 ± 0.04
Norharman	251 ± 3	229 ± 1	176 ± 4	0.45 ± 0.07	0.35 ± 0.02
Trp-P-2	ND	ND	ND	ND	ND

^a^ Note: heterocyclic aromatic amines (HAAs) were detected using LC-MS. ^b^ Not detected (ND).

**Table 2:** Changes in basic physical and chemical indexes of sesame seed oil refining process.

Oil Sample	Acid Value	Peroxide Value
	(mg/g)	(mmol/kg)
Crude oil	1.7 ± 0.0 ^a^	0.037 ± 0.00 ^b^
Degummed oil	1.6 ± 0.0 ^b^	0.069 ± 0.00 ^a^
Deacidified oil	0.24 ± 0.02 ^c^	0.024 ± 0.00 ^c^
Bleached (3% blended decolorizer) oil	0.29 ± 0.04 ^c^	0.037 ± 0.00 ^b^
Deodorized oil	0.28 ± 0.04 ^c^	0.003 ± 0.00 ^d^

Note: The letters ^a^, ^b^, ^c^ and ^d^ represent the differences among crude, degummed, deacidified, bleached and deodorized oils; the same letter indicates no significant difference (*p* > 0.05), different letters indicate a significant difference (*p* < 0.05).

**Table 3:** Changes in color of sesame seed oil during the refining process.

Oil Sample	L*	a*	b*
Crude oil	84.64 ± 0.02 ^a^	−9.29 ± 0.22 ^a^	16.55 ± 0.03 ^a^
Deacidified oil	86.20 ± 0.01 ^b^	−6.42 ± 0.02 ^c^	8.24 ± 0.03 ^c^
Deacidified oil	86.20 ± 0.01 ^b^	−6.42 ± 0.02 ^c^	8.24 ± 0.03 ^c^
Bleached (3% blended decolorizer)	89.68 ± 0.02 ^c^	1.69 ± 0.01 ^d^	−3.88 ± 0.06 ^d^
Deodorized oil	88.77 ± 0.01 ^c^	1.72 ± 0.02 ^d^	−4.47 ± 0.01 ^d^

Note: The letters ^a^, ^b^, ^c^ and ^d^ represent the differences among crude, degummed, deacidified, bleached and deodorized oils; the same letter indicates no significant difference (*p* > 0.05), different letters indicate a significant difference (*p* < 0.05).

**Table 4:** Qualitative and quantitative characterization of 14 HAAs with internal standard and optimized mass spectrum parameters.

HAAs	Precursor Ion [M + H]+ (*m*/*z*)	Diagnostic Productions (*m*/*z*)	Cone Voltage (V)	Collision Voltage (eV)	Dwell Time (ms)
AαC	184.0	167.2	108	32	30
		140.0	108	32	
MeAαC	198.2	154.1	104	40	30
		127.1	104	45	
Trp-P-1	212.0	168.0	80	30	30
		195.2	80	40	
DMIP	162.9	147.3	90	45	30
		105.0	90	45	
Glu-P-2	185.2	131.1	80	40	30
		78.2	80	40	
MeIQ	213.1	198.0	100	35	30
		144.0	100	60	
MeIQx	214.1	199.0	100	40	30
		131.0	100	55	
IQ	199.1	184.0	100	40	30
		157.0	100	50	
PhIP	225.3	210.2	120	45	30
		183.2	120	50	
4,8-DiMeIQx	228.1	211.8	100	45	30
		160.0	100	40	
7,8-DiMeIQx	228.0	131.3	100	55	30
		213.2	100	40	
4,7,8-DiMeIQx	242.0	227.1	120	40	30
		145.0	120	50	
Harman	183.0	115.0	120	50	30
		168.3	120	40	
Norharman	169.2	115.0	100	45	30
		142.0	100	40	
Trp-P-2	198.0	154.0	60	40	30
		128.0	60	40	

## Data Availability

Not applicable.

## Sample Availability

Samples are available from Wei Liu on valid request.
